# Perceptions of consent, permission structures and approaches to the community: a rapid ethical assessment performed in North West Cameroon

**DOI:** 10.1186/1471-2458-14-1026

**Published:** 2014-10-02

**Authors:** Jonas A Kengne-Ouafo, Theobald M Nji, William F Tantoh, Doris N Nyoh, Nicholas Tendongfor, Peter A Enyong, Melanie J Newport, Gail Davey, Samuel Wanji

**Affiliations:** Department of Microbiology and Parasitology, University of Buea, PO Box 63, Buea, Cameroon; Research Foundation in Tropical Diseases and Environment, PO Box 474, Buea, Cameroon; Department of Sociology and Anthropology, University of Buea, PO Box 63, Buea, Cameroon; Department of Sociology, University of Douala, Douala, Cameroon; Wellcome Trust Centre for Global Health Research, Brighton & Sussex Medical School, Falmer Campus, Brighton, BN1 9PX UK

## Abstract

**Background:**

Understanding local contextual factors is important when conducting international collaborative studies in low-income country settings. Rapid ethical assessment (a brief qualitative intervention designed to map the ethical terrain of a research setting prior to recruitment of participants), has been used in a range of research-naïve settings. We used rapid ethical assessment to explore ethical issues and challenges associated with approaching communities and gaining informed consent in North West Cameroon.

**Methods:**

This qualitative study was carried out in two health districts in the North West Region of Cameroon between February and April 2012. Eleven focus group discussions (with a total of 107 participants) were carried out among adult community members, while 72 in-depth interviews included health workers, non-government organisation staff and local community leaders. Data were collected in English and *pidgin*, translated where necessary into English, transcribed and coded following themes.

**Results:**

Many community members had some understanding of informed consent, probably through exposure to agricultural research in the past. Participants described a centralised permission-giving structure in their communities, though there was evidence of some subversion of these structures by the educated young and by women. Several acceptable routes for approaching the communities were outlined, all including the health centre and the *Fon* (traditional leader). The importance of time spent in sensitizing the community and explaining information was stressed.

**Conclusions:**

Respondents held relatively sophisticated understanding of consent and were able to outline the structures of permission-giving in the community. Although the structures are unique to these communities, the role of certain trusted groups is common to several other communities in Kenya and Ethiopia explored using similar techniques. The information gained through Rapid Ethical Assessment will form an important guide for future studies in North West Cameroon.

## Background

The informed consent process involves an education and information exchange that takes place between researchers and potential participants resulting in a decision about whether to take part in a given piece of research or not [1, 2]. Voluntary informed consent is universally accepted as a precondition for scientific research involving human beings. National and international guidelines for ethical conduct in research outline specific requirements for obtaining informed consent [1–4].

However, when externally sponsored research is conducted in low-income countries, several issues may arise when consent is sought from potential participants. In some communities it is usual (though not in line with national and international guidelines for ethical conduct in research) for male members of the family to make decisions on behalf of wives and adult children [5–10]. In other settings, community leaders, chiefs or elders take on a ‘gatekeeping’ role and determine access to whole communities by researchers [6, 8, 11, 12]. However, the decision of the community leader or family head should not replace individual consent [13].

In addition, research participants may not have the scientific literacy to understand information sheet templates developed in different cultural contexts [14, 15], thus the way in which information on the potential risks and benefits of research is provided must be refined. Researchers must aim to communicate information accurately and in an intelligible way, bearing in mind local knowledge and beliefs. It is recommended that information is given in simple, uncomplicated language over an appropriate period of time [16, 17]. Questions exist about the type of documentation that is suitable for use in low-literacy communities [18–20]. In these communities, it may be inappropriate to ask participants to sign consent forms, and witnessed verbal consent may be used instead [21].

Rapid ethical assessment is a brief qualitative intervention designed to map the ethical terrain of a research setting prior to recruitment of participants. It has been proposed as one means of improving the consent process in research-naïve settings [22, 23]. Rapid ethical assessment was carried out in Ethiopia prior to a genetic study to investigate the genetic basis of podoconiosis [5]. Such an intervention has never previously been used in Cameroon, yet findings by Tekola *et al*., and the paucity of information on informed consent within Cameroon prompted us to undertake rapid ethical assessment prior to a study aimed to further investigate genetic susceptibility to podoconiosis in the North West Region. We aimed to explore ethical issues and challenges associated with doing research in North West Cameroon, and in this article, we describe community and health professional perceptions surrounding informed consent, authority structures and approaches to the community. We believe that the outputs of this study will be useful to future researchers planning biomedical research in Cameroon.

## Methods

### Study area

**Figure 1:**
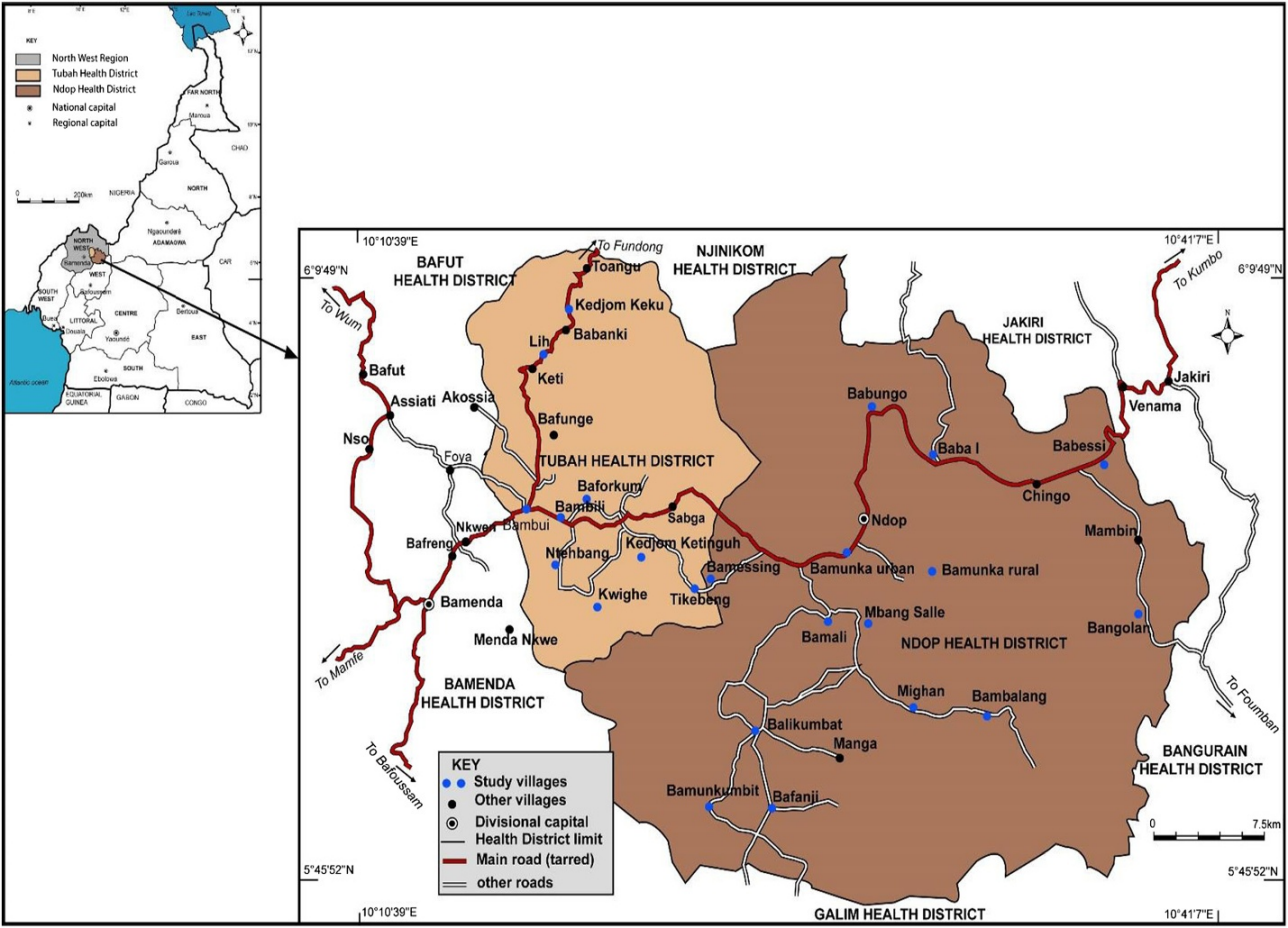
**Map of the study site.**

The study was carried out in Tubah and Ndop health districts of the North West Region of the Republic of Cameroon (Figure 1), where podoconiosis has been shown to exist [24, 25]. Tubah health district is found in Tubah Sub-division, located between longitude (10° 14′ and 10° 15′E and latitude 5° 06 and 6° 02′N). It has an average altitude of 1403 meters above sea level and covers an area of 340 kilometres square. Nine health areas in the Tubah health district were visited; Bambili, Tikebeng, Ntehmbang, Baforkum, Kedjom Ketiguh, Lih, Kwighe, Bambui, Kedjom Keku. Ndop health district is found in the Ngoketunjia Division which occupies much of the Ndop plain and is located between longitude 10° 20′ and 11° 25′E and latitude 5° 50′ and 5° 59′N. It has an average altitude of 1246 meters above sea level and covers a surface area of about 1154 Kilometres square. The health areas visited in the Ndop health district include: Baba I, Babessi, Babungo, Bangolan, Mighan, Bamessing, Bamunkumbit, Balikumbat, Bafanji, Bangsalle, Bamali, Bambalang, Bamunka rural and Bamunka Urban. All study communities in the two health districts have the rich volcanic soil often associated with podoconiosis, and the inhabitants mostly farm barefoot thereby exposing themselves to the disease. The communities under study are essentially agrarian and are involved in subsistence agriculture where basic food crops are produced for local consumption as well as for other national sub-regional markets.

### Study design

Data collection for this study was conducted from February to April 2012. Surveys were carried out in the study area, during which In-Depth Interviews (IDIs) and Focus Group Discussions (FGDs) were conducted. These were done using semi-structured interview guides adapted from a rapid assessment method validated in The Gambia [22, 23] and Ethiopia [5]. Three different interview guides were developed for IDIs and used as follows: one for health workers, one for researchers and the last one for community members, community leaders, and non-government organisation (NGO) members. A single FGD guide was prepared and used with young (aged between 18 and 25 years) and adult (>25 years) men and women living in the communities. The guides were prepared in English but administered in both English and Pidgin Lingua franca, an English-derived language commonly spoken and understood in the Anglophone parts of Cameroon. Sampling and recruitment were done until the data showed no new themes [6]. The interview guides were tested in Ndop and Tubah health districts with health workers and community members in the pilot phase of the study. In view of the low literacy levels of the population, informed oral consent was obtained from the study participants and digitally recorded before conducting the interviews and discussions. Discussions and interviews were conducted in settings that ensured participants’ privacy. Approval was given by the Cameroon National Ethics Committee and the Research Governance and Ethics Committee of Brighton & Sussex Medical School. This paper was written in accordance with the qualitative research review guidelines (BioMed Central editorial policies).

### Study participants

A purposive sampling strategy was used for the recruitment of health workers, NGO members and community leaders, while stratified random sampling was used for the recruitment of community members based on pre-defined inclusion criteria for enrolling participants. Using semi-structured interview guides, IDIs were conducted with 72 study participants in the Ndop (38 males and 7 females) and Tubah (20 males and 7 females) health districts. The disparity in the number of males and females was due to the fact that most health workers, community leaders and NGO members were men, and women were mostly interviewed as community members. In Depth Interview participants were grouped into four categories: i) health workers (n = 12), ii) Non-Government Organisation (NGO) leaders (9) and NGO members (11), iii) community members (23), community leaders (14), traditional ruler (1) and local government officer (1), and iv) a research scientist (1). All NGOs were cooperative groups involved in agricultural activities in the study area. In Depth Interviews lasted from 20 to 59 minutes. Eleven FGDs were conducted with a total of 107 participants, 6 with males and 5 with females in the two health districts. Focus Group Discussions were conducted with 30 young and 20 adult males, and 24 young and 33 adult females. The number of participants per FGD ranged between 6 and 12 persons. Most FGDs lasted between 35 to 50 min.

### Data collection

Data were collected by social scientists through direct interviews alongside a PhD student working on the genetic basis of susceptibility to podoconiosis. After explaining the nature and the objectives of the work to potential participants, those who consented took part in the interview. The interviews and FGDs were recorded with dictaphones with the permission of the participants. All social scientists involved in this work had previous experience in social science research in rural communities. However, before data collection, a working session was organized with the research assistants during which the nature and the objectives of the study were explained, and the IDI and FGD guides were studied, so that all investigators became familiar with the interview instruments and interviewing techniques.

### Data analysis

Data were collected in English and *Pidgin.* Interviews were transcribed anonymously, and interviews conducted in *Pidgin* were translated into English. Translation was done by experienced social scientists. To check for consistency, some of the interviews collected in *Pidgin* were translated twice by different people and the two English versions compared. Comparison showed good agreement. Close reading of the text was followed by coding based partly on themes identified in earlier research, and partly allowing themes to arise *de novo*. An inductive approach was used with themes of the latter type. The main themes addressed in this article are: perceptions of informed consent, authority structures in these communities, routes and strategies for approaching the communities, and factors facilitating or hindering recruitment to research.

## Results

A total of 179 participants (70 females and 109 males) took part in the study, the majority of whom were farmers. The age of the interviewees ranged between 18 and 78 years. The majority of fieldworkers and NGO leaders had attended secondary school. Most of the health workers had received their training in higher institutions, while most of the adult community interviewees had had no formal education.

### Perceptions of informed consent

Many of the communities had been exposed to agricultural research in the past. This, coupled with involvement in health research linked to widespread campaigns against HIV/AIDS, other sexually transmitted infections and malaria, meant that many community members had some understanding of consent. Community members described informed consent as a condition of knowing about the research prior to participation:

*-They [researchers] encourage us to know what their research or program is all about. If we are aware of that, we can participate and listen to everything they say (IDI-Community member female adult-Tubah)*

*-I think to come and take part in this work; we need to know the reason of the study; to know the objectives, and to be sure that you people are doing the right thing. (FGD with adult female community members-Ndop)*

Another community member viewed consent as a decision:

*-Consent is an issue that each person is supposed to take that decision within his family, like the head of the family is to decide what he wants with his family; it is not an issue that the village should decide. (FGD with adult male community members-Tubah)*

This view was echoed by a researcher who explained his view of informed consent in terms of a free decision to either participate in research or not:

*-…the purpose of a research consent is to let the participant know his rights, accept or not accept to participate in the project, if he accepts he participates but if he does not accept you don’t force a patient to participate in a research project. (IDI-Scientist in Tubah)*

With this degree of awareness, several respondents declared high degrees of willingness to consent:

*-Yes, I will love signing the document that has been presented to me because I have much interest in what you people are doing in this community today. (FGD with adult male community members-Ndop)*

### Authority structures in the communities

The communities involved in the study are mostly centralized systems with a central leader referred to as the *fon* (local traditional authority). He wields power on most but not all issues in the community. He is said to be the materialisation of the invisible, that is, the ancestors and as such he is highly respected in the society. The *fon* plays a very important role in the consent process, and most of the participants favoured the argument that consent for a biomedical research should first be given by the *fon*. This is clearly shown in the response given below by a health worker:

*- There is no lie I will tell you, people rely on the traditional council and the traditional council is under the fon. So when you contact the fon and the traditional council, the people will know that it is something good. (IDI-Health worker-Ndop)*

This response was further elucidated by the researcher who had been working in this area for some years. To him the *fons* constituted a very influential class of people who could not be by-passed as far as issues of consent were concerned*.* He stated:

*-…actually let us be fair to the communities. These are communities where the fons, the chiefs, are very much respected and they tend to believe and accept the fact that the community leaders are leading them in a good direction, so when the community leaders say ‘please take part in this research’ they tend to accept. (IDI-Scientist - Tubah Health District)*

Despite the sovereignty of the *fon*, some community members held the view that what concerns their families should remain the responsibility of the family head. Some participants maintained that they still had the freedom to choose what they want including consent to either participate or not in a study:

*-The family head should give consent because he is the custodian of the family and if anything happens to any member of the family he is the one to answer any questions that will come after it. (IDI-Male Community member-Ndop)*

*-If for example you enter my compound, you will get the permission from me as family head. Permission can also be obtained from the fon of the village, then the quarter head (the person heading the village quarter who is answerable to the fon and reports to him as the need may arise). (IDI-Adult male community member-Tubah)*

Adult community members considered family heads to be key players and to act as the gate keepers to their families, as captured in this argument by a community member:

*-Yes I think what he has said is correct that the family head should sign the consent because if we leave this decision to be taken by each individual in the family, when a problem will occur it will be the problem of the father who is the head to solve. Questions will be addressed but to you [the father] and not to the others. (FGD with Adult male community member-Tubah).*

The role of women in giving consent was not mentioned by many participants, as most women advocated for male responsibility in giving consent for research:

*-I think the father of the compound should be the one to give the authorisation for any of us in the compound to participate in any research like this… (FGD with adult females-Tubah)*

*-This is simply because the father is the family head and he is the one looking after all of us in the compound. (FGD with adult females-Tubah)*

*-She is correct saying the father is the one that should take the decision because if anything happens to us they will ask but him. (FGD with adult females-Tubah)*

Some women expressed the opinion that the decisions taken by men are often not correct. However, they noted that with negotiation the men may be persuaded to take another standpoint, or their decision may even be overruled:

*-No, not always, for us, it is clear that it is the father who always takes the decision, but that does not mean that he is always right. Sometimes, the father may say ‘no’ but after arguing with him (convincing him), he can change his decision. (FGD with adult females-Tubah)*

*-Sometimes when the family head’s decision is not satisfactory you can decide yourself whether to participate or not. (FGD with adult females-Tubah)*

Young people (under 25 years) held contrasting views on who should take responsibility for consent, being of the opinion that as adults they too had the right to take major decisions, since they were capable of distinguishing right from wrong and were as knowledgeable on these subjects as their parents. Male youths in a FGD in Tubah health district wanted to make their own decision about whether or not to participate in such research:

*-I don’t think somebody should decide for me now, so anything concerning consent or research should be determined and decided by me because I am old enough now to know what is good for me. (FGD with young males-Tubah)*

*-Somebody should not force you to go where you don’t like as we are not children, so we take decisions we know are correct and assume the responsibility since it is in taking a decision that you are aware of where you are going to and for what reason. (FGD with young females-Tubah)*

### Approaching the community

*Routes into the Community*. Several possible routes of entry were raised, including the *fon*, the quarter heads and the health centre. Most community members recommended the *fon* as the entry point, once clearance from the health system at Regional level was given. Health workers pointed to previous programmes requiring community access to illustrate the routes used:

*-It depends on the age group, for example, for the vaccine that we are giving, we have a target but when we don’t have a target group, we will just go directly to the fon and see the quarter head and explain the objective of the work to him. He will then give an announcement to invite people… We usually pass on the information from churches, social mobilisers, Njangi [solidarity fund raising group or local name for credit association] houses. (IDI-Health worker-Ndop)*

*-To approach the people – introduce yourself to the Health Centre or village authorities who will link you to the community. (IDI-Health worker -Ndop)*

These two apparently different routes are reconciled by other health workers who note that in order to access the *fon*, it is important first to communicate with the health centre:

*- When there is information to be passed on, the chief of centre conveys the message through the health committee members who meet the fons then the quarter head, then the community members. This is how the information circulates. (IDI-Health Personnel-Bambalang-Ndop)*

*-The health centre sends the message to the palace which then sends town criers that move across the village with the information giving the date and the time and requesting the people not to go to the farm on that day. (IDI-Health Personnel-Bambalang-Ndop)*

For the *fon*’s permission to be disseminated it passes through the counsellors and quarter heads to family heads. This process was described by several participants as being very important if any research is to succeed in the communities. In addition to obtaining informed consent for research, this same channel is often used to pass on any important piece of information (such as dates for National Immunization Days) to the communities. An adult male community leader traced this path:

*-Information can reach the community if it passes through the “fon”, Quarter Head, and Health Centre. (IDI-Community leader-Ndop)*

### Sensitisation processes

The importance of careful sensitization was emphasised by health workers. The recruitment of participants, they noted, was a very delicate task, and time and effort should be put in place to realize any such activity. With good sensitization, using accurate information, the study would have very high chances of succeeding:

*-The very first thing is sensitization. Before you invite somebody to participate in research, you have to inform the person, you have to make that person know what you are out for and what is the interest to him, to the community and the whole place in general. This should be done with the support of the health committee members who pass on the information to the community members. (IDI-Health worker-Ndop)*

*-To make them participate, I need to know the programme. When I know the programme, I also let them know about it and its importance. I pass through the traditional council to make sure that there is enough sensitization for them to understand so that most of them will make the right decision. When I do like that about 97% of the population participates. Only few will refuse (IDI-Health worker-Bamessing-Ndop)*

*-For publicity, advertisement, we pass through churches, we make announcement in markets, and we meet the quarter head through health committee members (IDI-Health worker Babessi-Ndop)*

### Information requested when approaching the community

Having shown fair understanding of consent, the participants raised very important issues as to the nature of information they would like before consenting to participate in a research project. Information on the nature of the research, the benefits of the research to the community and the objectives of the research were rated the most important among community members:

*-The information is this: This is research and research is to find out something about a particular disease and it is of great benefit to the community. So generally, you explain the objectives, why the research is being carried out and what is expected from the research. (IDI-Health worker Baba I Ndop)*

*Importance of the study to the community and what the study is all about. (IDI-Adult male Field worker-Tubah).*

However, respondents emphasized the “benefits” but did not mention the “risks” research may inflict on the participants, thereby underestimating the potential risks attached to biomedical studies.

### Delivery of information

It is vital that the objectives and nature of a study are fully explained before a participant is asked for consent. Many respondents pointed to the potential difficulties in conveying this information given low levels of literary and scientific understanding in the rural areas. Many potential participants might lack the ability to read written information sheets of consent forms, and those that read might have difficulties interpreting the information contained in the document. Many community members stated they preferred that the consent was read to them:

*-For me I will prefer that the expert reads out the information and takes time to explain to me; thereafter I will sign the document and keep it for reference when need be. This is very important for us because we are villagers who are not very knowledgeable on these aspects. (FGD with adult male community members-Ndop)*

A summary of the main findings per theme obtained in this study is presented in Table 1.

**Table 1:** **Summary of the main findings of the paper per theme developed**

Themes	Findings
**Perceptions of informed consent**	-Many community members had some understanding of consent. They described informed consent as a condition of knowing about the research, and making a decision based on this knowledge.
	-This knowledge of consent was attributed to their previous involvement in agricultural as well as health research (widespread campaigns against HIV/AIDS, other sexually transmitted infections and malaria)
**Authority structures in these communities**	-Community leaders (*fons, quarter heads*) and family heads were considered to play an important role in the informed consent process.
	-The *fon* was said to be the materialisation of the invisible, that is, the ancestors and as such, was highly respected in the society.
	-The *fons* constituted a very influential class of people who could not be by-passed as far as issues of permission were concerned.
	-However, these findings were not in line with national and international guidelines for ethical conduct in research which recognize that whatever the cultural context within which research is conducted, individual informed consent should be given voluntarily by competent participants involved in the study and not by others.
	-In our cultural context, researchers are therefore recommended to obtain permission to approach from community leaders (*fon, quarter heads)* and family heads before seeking individual informed consent.
**Routes and strategies for approaching the communities**	- Several possible routes of entry were raised, including the *fon*, the quarter heads and the health centre. Most community members recommended the *fon* as the entry point, once clearance from the health system at Regional level was given (Figure 2).
	-Health workers were seen to be very important in the sensitization process; however they themselves must be clear about the nature of informed consent and the distinction of this process from one of maximising recruitment.
**Factors facilitating or hindering recruitment to research**	-Approach to the community: contacting the community leaders and family heads may facilitate the recruitment process
	-Involvement of health staff in the sensitization process may also facilitate both the informed consent and recruitment process. However the health staff must be familiar with the research protocol.

## Discussion

According to Marshall *et al*. [26], informed consent depends upon an individual’s accurate understanding of the nature and purpose of the study including its potential risks. Responses by community members in both Tubah and Ndop health districts suggested they could indeed identify two key facets of consent: firstly, ‘knowing’ (i.e. being informed) about a study, and secondly, making a decision based on this knowledge. This understanding of consent may be explained by previous involvement of the community in agricultural studies, and contrasts with studies in communities in Kenya and Ethiopia [5, 14, 27]. In Kenya, consent was considered to be potentially prejudiced by misunderstandings of the specific research proposed [27], while in southern Ethiopia, assumptions that information provided prior to studies was health education weakened communities’ understanding of consent [5].

Respondents were generally clear about the information they would like in order to give consent. This included not simply ‘what the study was about’ but also its likely impact (‘the importance of the study to the community’). Several pointed out the difficulties of conveying this information given low levels of literacy, but were able to come up with strategies to overcome this, including having information read out, a researcher spending time talking it through, and potential participants being given a written summary to keep for further discussion with literate family members. These approaches were very similar to those suggested in a low-literacy community in southern Ethiopia [5]. As many have previously observed [6, 28–30], low literacy does not mean the inability to understand issues of consent.

Respondents did not specifically mention a desire to know about the potential risks related to research. They seemed to be more interested in the benefits and importance of the research and other issues such as confidentiality and duration of research than the potential related risks.

Nevertheless, they emphasized that they wanted to be given full information about the research. Underestimation of the potential risks attached to research, particularly for a clinical trial or genetic research, would weaken the informed consent process [1–4]. Informed consent presupposes that subjects are well informed about the study, the potential risks and benefits of their participation and that it is research, not therapy, in which they will participate [31]. More studies aiming to investigate the perceptions of community members about the potential risks they think could be associated to research are encouraged in our setting.

In most of the communities the participants stated they were comfortable to give written consent. This may be because these communities have been influenced by major educational institutions in the North West region, creating awareness of the value of documentation. This finding is contrary to that of Tekola *et al*. [5] who found that respondents in Wolaita Zone of Ethiopia did not favour the use of signed consent forms. The context of the Tubah and Ndop communities is different because the people have a culture of signing documents during small scale financial transactions in their *tontines* (credit associations).

The voluntary nature of the decision in consent was touched upon by community members, and more clearly identified by the researcher interviewed, who mentioned patients’ rights and the importance of not using force in relation to the consent process. Nelson and colleagues recently described a model of voluntariness in consent which included internal and external influences and constraining situation [32]. This has been refined by Kamuya *et al.* [27], and the importance of contextual factors further emphasised. We explored the potential of the major factors identified by Kamuya *et al.* (including understanding research, social norms and social relationships) to influence voluntariness of consent processes in North West Cameroon.

Exploration of authority structures and approaches to the community showed that though most participants were accustomed to the principle of consent, many were not giving consent independently. Voluntariness was affected by social relationships, in that many respondents indicated that they did not take decisions themselves, but were subject to decisions taken on their behalf either by the community leader (*fon*) or at a closer level by their family head, most often a husband or father. This has been widely reported in other contexts, including in Japan, Nigeria, Botswana and China [6–12]. Proxy decision-making is not in line with national and international guidelines for ethical conduct in research which recognize that some standards, such as that requiring individual informed consent be given voluntarily by competent participants, be met whatever the cultural context within which research is conducted [1–4, 13, 31]. In the communities studied, proxy decision-making may be closely tied in with trust in existing hierarchies, which under some circumstances may contribute to the best interest of the potential participant, but under others may generate serious ethical issues [6, 29, 33]. In such situations, the chief’s capacity to critically appraise a research proposal is key to avoiding community exploitation, and this capacity cannot be taken for granted. In the present cultural context, it would be advisable for researchers to seek permission from the chief or quarter head or from the family head before individuals are approached for individual informed consent [13]. Further studies are encouraged, to explore whether people follow elders’ instructions solely because of the trust they place in them, or because of fear of being dismissed from social gatherings and other community activities [5]. Although men (family heads) were declared to have the dominant role in making decision, it should be pointed out that the father was also acknowledged as the person who should give permission because he is the one who will take action if something goes wrong. Thus, what appears at first sight as just paternalism or a rigid family structure may also be linked to the concept of “responsibility, concern and love” [6].

However, some focus group discussions suggested that this social norm was being questioned. Two sub-groups, women and young people, raised arguments against decisions being taken on their behalf by household heads. Some women doubted that a household head’s decision was always correct, and others considered these decisions to be negotiable. Young men (mostly students back in their communities during semester breaks) took the arguments further, suggesting it was patronising that decisions were still taken on their behalf once they were “*old enough now to know what is good..*”.

**Figure 2:**
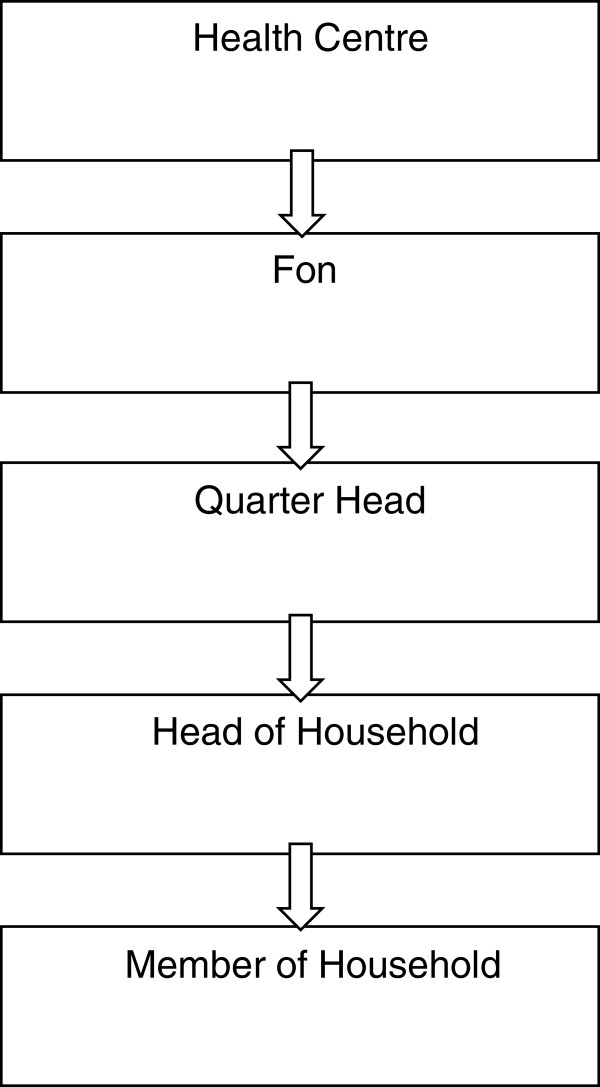
**Approach to Communities in Tubah and Ndop Health Districts, North West Cameroon.**

While not all agreed that community leaders should take decisions on their behalf, most saw the role of the fon in liaising with the different stakeholders in the community as crucial to those wishing to gain access to the community for research. The fon was portrayed as the custodian and facilitator of engagement with the community. The three groups involved in accessing communities for research were identified as community leaders (fon and quarter heads), the formal health system (often referred to as the ‘Health Centre’), and the research team. The health system was considered to facilitate initial links between the research team and community leaders, and the community leaders then permitted dissemination of information to household heads through traditional council meetings, town criers, churches and other community members for better understanding of the message (Figure 2). Health workers repeatedly mentioned thorough sensitization through the traditional council, churches and health committee members as being vital to successful recruitment.

The importance of inter-personal and institutional trust in approaching a community for consent for research in a low-income setting has been described in Kenya and Ethiopia. Around Kilifi, Kenya, inter-personal relationships with the study team were demonstrated to be important [34], as has also a foundation of long-standing trust in the Kenyan Medical Research Institute (KEMRI) [35]. In southern Ethiopia trusted gatekeepers included a patient association rather than traditional leaders [36]. In this study in North West Cameroon, where there is no patient association and health research is not as well established as in Kilifi, the trusted groups are community leaders and the formal health system. It is therefore vital that members of these groups have a clear understanding of the nature of informed consent. Comments by a health worker in an in-depth interview suggested that he saw his role as maximising participation rather than facilitating a decision-making process. Training of members of both groups is clearly important if the trust placed in each is not to be misused.

There are several limitations to this study. Although a large number of in-depth interviews were conducted, women were under-represented, partly because community leaders and health workers were more likely to be men and partly because data collection was performed during the peak farming season when women in particular found it difficult to leave farming activities. Only one researcher was interviewed. Despite efforts to minimise social desirability bias, responses may reflect what interviewees thought the researchers wanted to hear rather than their true opinions. Although interviews were conducted in both English and Pidgin, analysis was performed in English, which may have masked some findings – qualitative studies analysed in the language of data collection often yield richer meaning.

## Conclusion

In summary, using rapid ethical assessment techniques, we have demonstrated a reassuring level of knowledge about consent in rural communities in North West Cameroon. Understanding about studies and about permission-giving appears to have arisen from previous exposure to agricultural research. Respondents were able to outline the structures of authority and permission-giving in the community, and clear routes by which communities might be approached. Although the structures are unique to these communities, the presence of certain, trusted groups is common to several other communities in Kenya and Ethiopia explored using similar techniques. The information gained through rapid ethical assessment will form an important guide for future studies in North West Cameroon.
